# Progress in Surgery

**Published:** 1895-11-23

**Authors:** 


### PROGRESS IN SURGERY.

### GENITO-URINARY SURGERY.

(*Continued from page* 119.)

**Tumours of the Bladder**—Mr. Henry Fenwick[23] has discovered only one hundred cases of hydatids of the bladder in the literature for two hundred years; even in Australia, where the disease is rife, such cases are but seldom encountered. The cyst usually lies between the bladder and rectum, under the peritoneum. The muscular and fascial floors of the pubes check the downward enlargement of the cyst, so that it enlarges upwards and outwards, pushing the peritoneum before it, and displacing also the bladder upwards and forwards, and seriously pressing on the ureters. So powerfully is the bladder pushed against the pelvis that it is often transformed into an hour-glass shape. The urethra is so dragged on as to be considerably lengthened, and catheterisation rendered extremely difficult or impossible. In one case in which Mr. Henry Fenwick operated he came upon a thick muscular body, which covered the front of the cyst. This was the empty hypertrophied bladder thrust bodily upwards. In the great majority of cases a very characteristic group of symptoms demonstrates the existence of a wedge between the bladder and rectum. The constipation produced by the pressure of the cyst is often considered of no moment, and the first symptom noticed is usually irritability of the bladder, which is soon accompanied by increasing difficulty in micturition, and terminates abruptly in complete retention. Mr. Bryant has recorded a remarkable case of hydatid cyst in this situation, in which retention, lasting a week, occurred on two occasions with an interval of seven years, and then seven years later again recurred, when Mr. Bryant drained the cyst, but the man died. A sacculated bladder may be mistaken for a cyst, but the use of the catheter would exclude such a condition. Sarcomata and syphilomata are rarer in the pelvic fascia even than hydatids. The characteristic fluid can be withdrawn on puncture, after thoroughly emptying the bladder first with a catheter. These cysts very rarely burst into the bladder and urgently demand incision and drainage, all the daughter cysts and as much of the inner wall as possible being removed. If left they cause death from pressure on the ureters. The operation Mr. Henry Fenwick considers should be extra-peritoneal above the pubes. If retention is present and a catheter cannot be passed, the cyst (not the bladder) should be aspirated, when usually the urine will flow, and then the cyst should be immediately opened and drained as in cases uncomplicated by retention, or suppuration is very likely to follow.

Chevalier[24] considers that with the exception of the trigone and the region about the ureters, extirpation of the entire bladder is possible. Partial extra-peritoneal resection of the anterior wall has been frequently and successfully practised. Various methods are described for dealing with the ureters if the portion of bladder into which they open are cut away. A method of removal of growths on the portion of the bladder covered by peritoneum is also described, in which the wall of the bladder is infolded so as to invaginate the growth into the bladder, and the growth then excised and the bladder filled with iodoform gauze. The peritoneal cavity is then opened and the peritoneal surfaces sewn together accurately.

Dr. Nitze[25] (Berlin) has performed suprapubic cystotomy for tumours of the bladder sixty-six times with a mortality of 17·5 per cent., and out of eighteen cases in which he sutured the bladder, thirteen had no leakage. The author considers that even benign tumours are very apt to occur after suprapubic cystotomy at places rather distant from the seat of the original tumour. He has operated in several cases by means of a snare, which crushes through the base of the growth, passed through the urethra and used with the cystoscope. The cut surface was then cauterised with the galvano cautery. The hæmorrhage as a rule was insignificant. Cocaine can be used instead of chloroform. Most of the patients operated on by this method were males. The tumours varied in size from a hazel nut to an apple. If cystitis supervenes it disappears in a short time after operation. Mr. Battle reports[26] a case of carcinoma which involved the female urethra and neck of the bladder, causing retention. The bladder was first opened above the pubes, and a drainage tube inserted, and then the urethra and growth which involved the neck of the bladder were removed, the opening in the bladder (which admitted three finger tips) being closed by sutures.

**Hernia of the Bladder.**—Treves[27] describes how he discovered the early formation of a hernia of the bladder, in operating for the radical cure of hernia. The rupture was small and easily reducible. On exposing the external ring he found a mass of fat protruding beneath the cord from below the peritoneum, and underneath this a small empty peritoneal sac, which was cut off. The removal of the fatty hernia revealed another and larger protrusion behind it. This was also of fat, but contained the summit of the bladder within it. This case Treves considers illustrates the traction theory of hernia. The increasing sub-peritoneal fat in the antevesical space extends along the inguinal canal and drags a portion of the bladder after it just as the fat extends from beneath the peritoneum, and drags a fold of peritoneum with it.

[23] International Clinics, Vol. IV., p. 227. [24] Therapeutic Gazette, March 15, 1895, p. 197. [25] The Medical Week, March 15, 1895. [26] Lancet, June 15, 1895, p. 1,512. [27] Lancet, June 8, 1895.

